# Neuropsychological Outcomes after Surgery for Olfactory Groove Meningiomas

**DOI:** 10.3390/cancers13112520

**Published:** 2021-05-21

**Authors:** Paul E. Constanthin, Renato Gondar, Julia Fellrath, Isaline Mottet Wyttenbach, Karima Tizi, Leo Weman, Pia Vayssière, Karl Schaller, Torstein R. Meling

**Affiliations:** 1 Department of Clinical Neurosciences, Division of Neurosurgery, Geneva University Hospitals, 1205 Geneva, Switzerland; paul.constanthin@hcuge.ch (P.E.C.); renato.gondar@hcuge.ch (R.G.); karima.tizi@hcuge.ch (K.T.); Pia.Vayssiere@hcuge.ch (P.V.); Karl.Schaller@hcuge.ch (K.S.); 2 Neuropsychology Unit, Department of Clinical Neurosciences, Geneva University Hospitals, 1205 Geneva, Switzerland; Julia.Fellrath@hcuge.ch (J.F.); Isaline.Mottet@hcuge.ch (I.M.W.); 3 Faculty of Medicine, University of Lausanne, 1011 Lausanne, Switzerland; leo.weman@hcuge.ch; 4 Faculty of Medicine, University of Geneva, 1206 Geneva, Switzerland

**Keywords:** surgery, neurosurgery, cognition, outcome, meningioma, neuropsychology

## Abstract

**Simple Summary:**

Olfactory groove meningiomas account for 9% of all meningiomas, present with unique molecular characteristics and are standardly treated by surgical removal. Due to their location (in direct contact with cortical structures exerting key-role functions in cognition), olfactory groove meningiomas might negatively impact cognitive abilities. However, little is known about the effects of both their presence and surgical removal on patients’ neuropsychological functions. In this retrospective study, we analyze the evolution of neuropsychological abilities in 17 patients benefitting from surgical removal of an olfactory groove meningioma. We observed that those patients already presented with preoperative deficits in pre-frontal cortex cognitive functions, more pronounced in the domain of cognitive flexibility, followed by attention. After an initial postoperative worsening, patients tended to improve in the majority of cognitive aspects, with attention and flexibility remaining impaired at one-year follow-up.

**Abstract:**

Background: In recent years, several studies have reported abnormal pre- and postoperative neuropsychological functioning in patients with meningiomas located in the prefrontal cortex (notably the ventromedial region). In the case of olfactory groove meningiomas, the tumor is in direct contact with the inferior aspect of the prefrontal cortex, a cortical region with crucial roles in decision-making, cognition and memory functions, potentially negatively impacting neuropsychological functions. Materials and Methods: We retrospectively compared pre- and post-operative neuropsychological testing of 17 patients undergoing surgical removal of olfactory groove meningiomas in our institution between January 2013 and December 2018. Neuropsychological results were obtained from the patients’ medical history and normalized as z-scores of their respective cognitive functions. Results: Assessment of cognitive follow-up showed an important heterogeneity among patients. Pre-operative cognitive impairment was observed in most patients, particularly in cognitive flexibility (mean z-score: −1.35). Immediate post-operative cognitive status showed an overall impairment in all domains of cognition, significant for the domains of attention (*p* = 0.0273) and flexibility (*p* = 0.0234) and almost significant for the domain of language (*p* = 0.0547). The late follow-up at one year showed a trend towards general improvement, although attention and flexibility remained impaired. Discussion: Olfactory groove meningiomas impact pre-frontal cortex cognitive functions, particularly in the domain of cognitive flexibility. After an initial postoperative worsening, patients tended to improve in most aspects after one year, aside from cognitive flexibility and attention.

## 1. Introduction

Meningiomas are among the most frequent intracranial tumor types, with an estimated annual incidence of approximately 8 cases per 100,000 [1]. In the vast majority of cases, they present a benign course [2], but meningiomas can become symptomatic (triggering symptoms such as epileptic seizures) due to the mass effect on the adjacent parenchyma or the associated edema [3]. The gold standard in meningioma management when lesion becomes symptomatic, shows radiological growth or is already compressive at the time of diagnosis, remains microsurgical resection [2,4,5,6,7,8], with radiotherapy [2], radiosurgery [9], or proton therapy [10] being considered for high-grade or atypical meningiomas (adjuvant) or in the case of recurrences [2].

Traditionally, the success rate of surgical removal for meningiomas has been evaluated on the basis of a combination of the intra-operative macroscopic removal of the lesion [4,5,6,7,8,11] (i.e., the Simpson grade [12]), post-operative imaging and resolution or improvement of associated neurological focal deficits or epilepsy. Regrettably, scientific publications assessing long-term post-operative health-related quality of life (HrQoL) and functional disability in patients after meningioma surgery are scarce and the data are heterogeneously reported [13].

However, in recent years, some studies have reported abnormal neuropsychological functioning in patients after meningioma surgery for tumors typically located in the frontal and prefrontal regions (notably the ventromedial or the medio-lateral region), in particular executive functions [14,15,16,17,18,19,20,21,22,23,24,25,26,27,28]. Executive functions encompass many interrelated higher-order cognitive processes, allowing people to take goal-directed actions [29]. The capacities to update and monitor working memory representations (updating), to inhibit impulses (inhibition), and to shift attention from one task set to another (flexibility) are considered in the literature as important executive function components [30].

Meningiomas of the olfactory groove originate from arachnoid cells of the lamina cribrosa and fronto-ethmoidal suture, in contrast to planum sphenoidale and tuberculum sella lesions, which arise from dural attachments posterior to the fronto-sphenoidal suture and anterior to the chiasmatic sulcus, respectively. They account for 7–18% of all operated intracranial meningiomas [7,31] and may differ from the ones in other locations at a molecular and epigenetic level [32,33,34]. Despite such singularities, little is known about their clinical consequences as past studies mostly focused on their effect on olfaction [35,36]. However, in the case of olfactory groove meningiomas where there is direct contact with important cortical cognitive areas such as the pre-frontal and rostral cortex, the potential for those tumors to affect neuropsychological functioning deserves special attention [14,17,22,23,24,25,26,27,28,35,37].

In this study, we retrospectively analyzed pre- and postoperative neuropsychological tests of patients undergoing surgical removal of olfactory groove meningiomas in our institution. We focused on six dimensions of cognition (i.e., 1—memory and orientation, 2—attention, 3—language, 4—inhibition, 5—updating, and 6—flexibility) and analyzed the impact of neurosurgical removal of olfactory groove meningiomas on the patients’ neuropsychological status.

## 2. Materials and Methods

### 2.1. Study Design and Patient Selection

After obtaining the approval from the local Ethics Committee (2019-02385) and in compliance with regulations, we retrospectively obtained the list of every patient that underwent a surgical procedure for removal of a meningioma in our institution between January 2013 and December 2018. Medical records, neuroimaging studies, and pathology reports were reviewed by two independent investigators (PEC and LW) to identify patients with olfactory groove meningioma or meningioma presenting with a significant mass effect on the olfactory groove. The senior investigator (TRM) would eventually decide in case of disagreements. A letter was sent to every patient operated on for an olfactory groove meningioma during the study period (*n* = 21), and they were given the right to refuse the re-utilization of their data in our study. A cohort of 21 patients was identified, of whom 17 had pre- and postoperative neuropsychological records for further analysis.

Patients were evaluated with gadolinium-MRI both pre- and postoperatively to assess the extent of resection and intra-operative Simpson grading [12] was available for each individual. The tumor volume was calculated with Brainlab software (IGS planning, iPlan 3.0 cranial; Brainlab AG, Feldkirchen, Germany). Fluid-attenuated inversion recovery (FLAIR) or T2 sequences were evaluated to measure the porencephalic cavity and edema (hypo- and hyper-intense changes) in frontal lobes and neighboring regions as well as the absence of a clear tumor–parenchyma delimitating interface (i.e., the absence of a clear delimitation between tumor edges and healthy cerebral parenchyma in T2/FLAIR and absence of a capsular frontier in the T1-gadolinium sequences). A recent systematic review [38] gives an update on the controversies of cortical invasion definition (as well as tumor–parenchyma interface) and highlights the different criteria utilized for this purpose in contemporaneous literature: radiological aspects, pathological differentiation and intraoperative assessment of adhesions between meningioma and healthy brain.

### 2.2. Neuropsychological Evaluation and Other Outcomes

Two neuropsychologists (IM and JF) extensively reviewed patients’ medical history and extracted all the available data regarding pre-, immediate postoperative and follow-up (i.e., more than 2 months after surgery) neuropsychological tests. However, patients were submitted to a total of 66 different neuropsychological assessment tools. To ensure comparability, the results of those tests were normalized as z-scores of their respective cognitive functions, in accordance the the “ASNP 2018 criteria” [39], and average z-scores were categorized for the six following cognitive dimensions: 1—memory and orientation; 2—attention, 3—language, 4—inhibition, 5—updating, 6—flexibility, as described in previously published works [19,29,30,40]. These were obtained for each patient at every possible time-point (preoperative, immediate postoperative and follow-up). Patients’ neuropsychological results for each category were also categorized as either “impaired” (i.e., average z-score < −1.6 or “clearly inferior to the norm”) or “non-impaired”. The value of −1.6 was selected as it represents the value at which neuropsychological tests are considered as significantly impaired and can therefore significantly alter the patients’ quality of life.

### 2.3. Statistical Analysis

GraphPad Prism version 8.0 (GraphPad, San Diego, CA, USA) was used for statistical analysis. After controlling for normal distribution by using Shapiro–Wilk normality tests, paired Wilcoxon tests (when distribution was not normal) or paired t-tests (when distribution was normal) were performed to compare z-scores. Fisher exact tests were used when comparing qualitative data (“impaired” vs. “non-impaired”).

### 2.4. Data Availability

All data generated and analyzed during this study are available from the corresponding author on reasonable request.

## 3. Results

### 3.1. Patient Demographics

**Figure 1:**
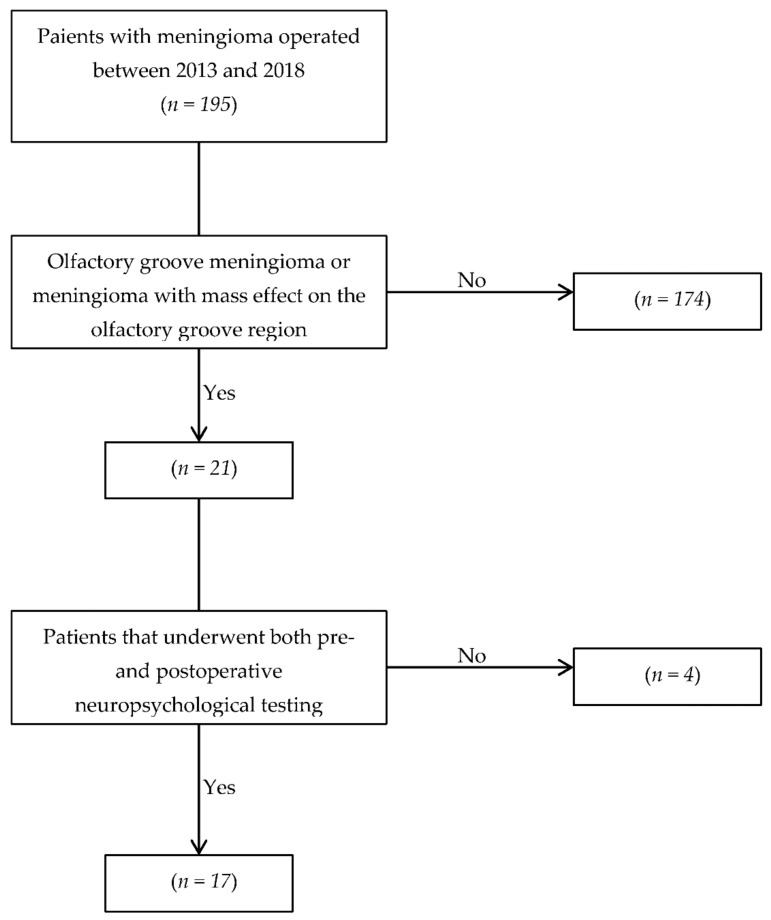
Patient selection flow-chart. This figure shows the process that we underwent to select our patients.

Seventeen patients who underwent surgery for olfactory groove meningioma during the study period had detailed neuropsychological tests pre- and postoperatively (Figure 1). The patients’ age ranged from 22 to 77 years (mean 58.7 years) with a comparable distribution between genders (nine male patients, 52.9% and eight female patients, 47.1%) (Table 1). All meningiomas were classified as grade I according to the World Health organization (WHO) brain tumor classification except one (classified as WHO grade II) [41], who benefitted from adjuvant radiotherapy accordingly to institutional guidelines and predominantly originated directly from the olfactory groove or planum sphenoidale. The relevant patient and meningioma characteristics are summarized in Table 1.

### 3.2. Mass Effect on the Olfactory Groove Results in Impaired Preoperative Cognitive Performance

**Figure 2:**
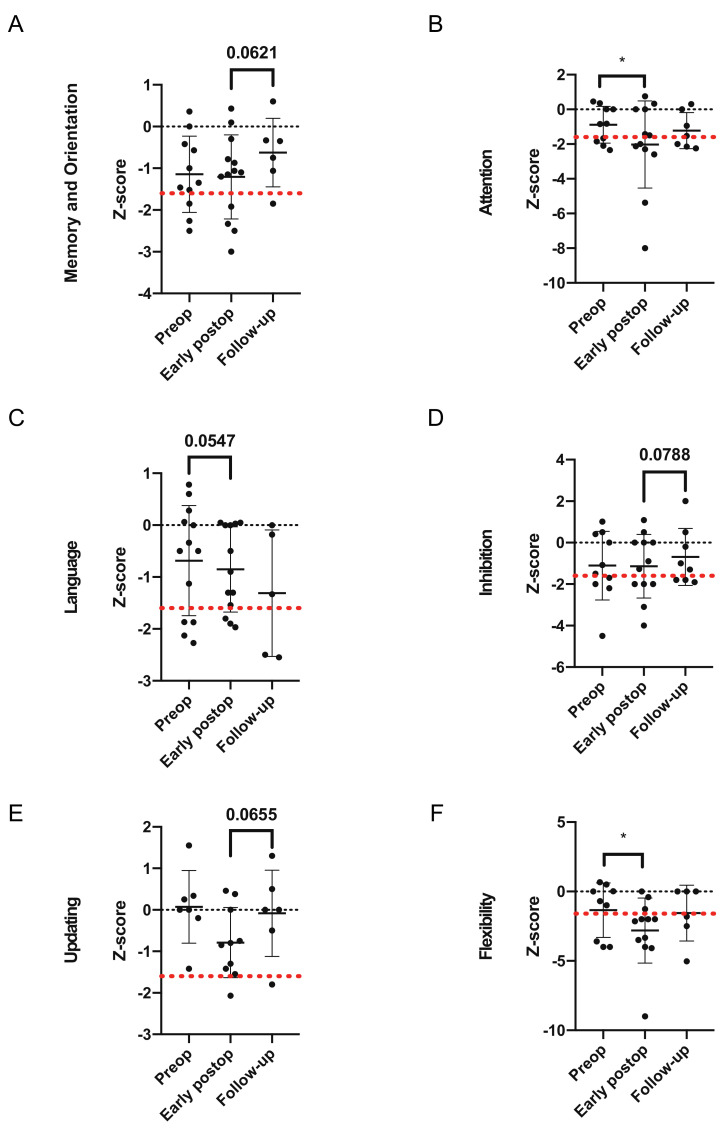
Evolution of patients’ normalized z-scores for the 6 selected neuropsychological categories. (**A**) Comparison of the normalized z-scores in the “Memory and Orientation” category of neuropsychological tests (preop = preoperative z-score; early postop = first z-score measured immediately after surgery; follow-up = second z-score measured within one year). (**B**) Comparison of the normalized z-scores in the “Attention” category of neuropsychological tests (preop = preoperative z-score; early postop = first z-score measured immediately after surgery; follow-up = second z-score measured within one year). (**C**) Comparison of the normalized z-scores in the “Language” category of neuropsychological tests (preop = preoperative z-score; early postop = first z-score measured immediately after surgery; follow-up = second z-score measured within one year). (**D**) Comparison of the normalized z-scores in the “Inhibition” category of neuropsychological tests (preop = preoperative z-score; early postop = first z-score measured immediately after surgery; follow-up = second z-score measured within one year). (**E**) Comparison of the normalized z-scores in the “Updating” category of neuropsychological tests (preop = preoperative z-score; early postop = first z-score measured immediately after surgery; follow-up = second z-score measured within one year). (**F**) Comparison of the normalized z-scores in the “Flexibility” category of neuropsychological tests (preop = preoperative z-score; early postop = first z-score measured immediately after surgery; follow-up = second z-score measured within one year). Paired Wilcoxon test (**B**,**C**,**F**) and paired t-test (**A**,**D**,**E**) between preop and early postop and between early postop and follow-up; error bars, SD; * *p* < 0.05; black dots indicate patients’ individual tests; red-dotted line stands for z-score = −1.6.

**Figure 3:**
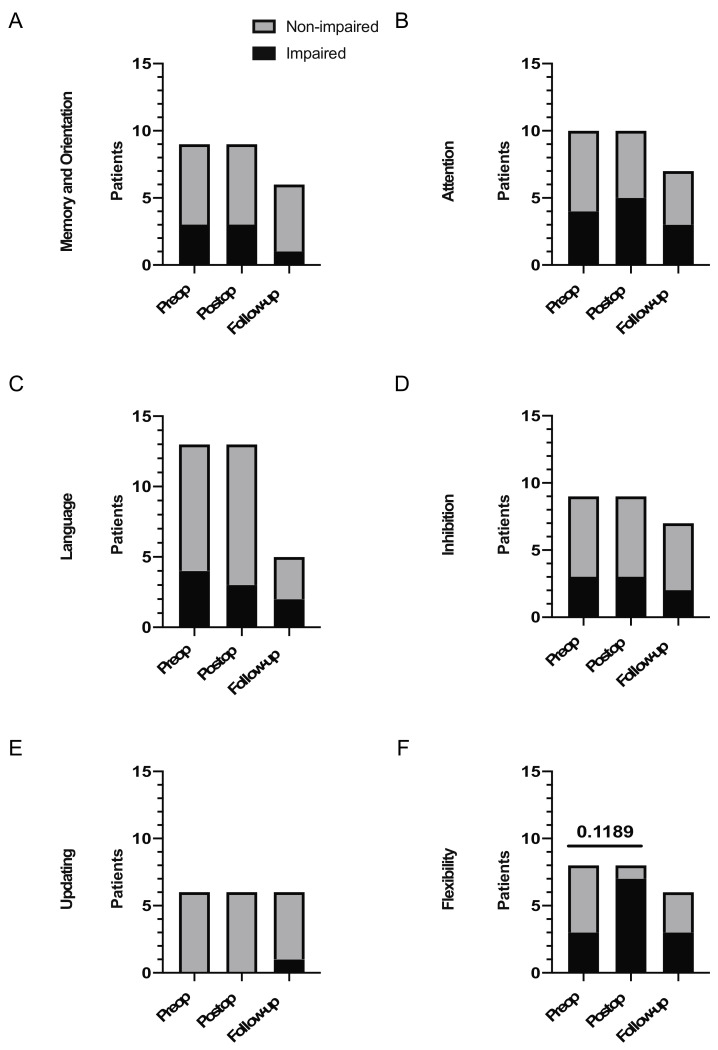
Comparison of the evolution of the proportion of patients with impaired z-scores for the 6 selected neuropsychological categories. (**A**) Comparison of the proportion of patients with impaired (z-score lower than −1.6) and unimpaired (z-score equals or higher than −1.6) normalized z-scores in the “Memory and Orientation” category of neuropsychological tests (preop = preoperative z-score; early postop = first z-score measured immediately after surgery; follow-up = second z-score measured within one year). (**B**) Comparison of the proportion of patients with impaired (z-score lower than −1.6) and unimpaired (z-score equals or higher than −1.6) normalized z-scores in the “Attention” category of neuropsychological tests (preop = preoperative z-score; early postop = first z-score measured immediately after surgery; follow-up = second z-score measured within one year). (**C**) Comparison of the proportion of patients with impaired (z-score lower than −1.6) and unimpaired (z-score equals or higher than −1.6) normalized z-scores in the “Language” category of neuropsychological tests (preop = preoperative z-score; early postop = first z-score measured immediately after surgery; follow-up = second z-score measured within one year). (**D**) Comparison of the proportion of patients with impaired (z-score lower than −1.6) and unimpaired (z-score equals or higher than −1.6) normalized z-scores in the “Inhibition” category of neuropsychological tests (preop = preoperative z-score; early postop = first z-score measured immediately after surgery; follow-up = second z-score measured within one year). (**E**) Comparison of the proportion of patients with impaired (z-score lower than −1.6) and unimpaired (z-score equals or higher than −1.6) normalized z-scores in the “Updating” category of neuropsychological tests (preop = preoperative z-score; early postop = first z-score measured immediately after surgery; follow-up = second z-score measured within one year). (**F**) Comparison of the proportion of patients with impaired (z-score lower than −1.6) and unimpaired (z-score equals or higher than −1.6) normalized z-scores in the “Flexibility” category of neuropsychological tests (preop = preoperative z-score; early postop = first z-score measured immediately after surgery; follow-up = second z-score measured within one year). Fisher’s exact test (**A**–**F**). No significant value.

Pre-operative neuropsychological testing revealed that the average z-scores for all categories were inferior to 0 in our patients except for the category of “updating”, suggesting at least some level of impairment in five out of the six tested categories (Figure 2A–F). Of note, the sixth category (flexibility) showed the lowest average z-score (−1.35) (Figure 2F), with the categories of “memory and orientation” and “inhibition” also showing z-scores inferior to −1 (−1.14 and −1.11, respectively) (Figure 2A,D). When dividing the patients into two groups, “impaired” and “non-impaired” (with patients in the impaired group showing a z-score lower than −1.6), the categories that showed the highest proportion of patients with “impaired” results were “inhibition” and “attention” with, in both cases, 40% of the patients showing impaired performance (Figure 3A–F).

### 3.3. Postoperative Neuropsychological Testing Shows Worsening of Cognitive Performance after Surgery

A comparison of z-scores from the different neuropsychological testing dimensions immediately after surgery (1 to 7 days postoperatively) showed a tendency towards a worsening in four of the six tested categories. Of those four categories, the differences in attention (category 2) and flexibility (category 6) were the only statistically significant findings (*p* = 0.0273 and *p* = 0.0234, respectively), while the difference in language (category 1) tended towards significance (*p* = 0.0547) (Figure 2A–F). These results were confirmed by categorical comparison, with the proportion of “impaired” patients increasing in the categories of attention and particularly flexibility, albeit not significantly when compared with the preoperative situation (Figure 3A–E). Of note, flexibility (category 6) showed, again, the worse average z-score (−2.81), with a statistical test that tended towards significance when comparing the number of ”impaired” patients before and after surgery (*p* = 0.1189, Figure 3F). This category and the one of attention were the only categories with an average z-score being categorized as “impaired”.

### 3.4. Longer-Term Neuropsychological Follow-Up Shows a Tendency towards Improvement in Some Parts of Patients’ Cognitive Performance

We finally analyzed neurological testing of patients after a long-term follow-up in our neuropsychological department and compared those data to the ones obtained in the early postoperative period. Interestingly, all neuropsychological categories but one showed improvement in their average z-score, with categories 1, 4, and 5 showing improvements that tended towards significance (*p* = 0.0621, *p* = 0.0788, and *p* = 0.0655 respectively) (Figure 2A–F). On the contrary, the third category (language) showed a non-significant tendency towards worsening (Figure 2C). Flexibility, again, showed the worse average z-score (−1.56).

## 4. Discussion

In our study of olfactory groove meningiomas, the objectives were to evaluate the cognitive consequences of these lesions, to identify possible predictive elements, and to discuss their subsequent treatments and follow-up. We observed that patients with a meningioma responsible for a mass effect on the pre-frontal and rostral cortical region adjacent to the olfactory groove show a tendency for lower performance in cognitive neuropsychological tests, particularly in the field of ”flexibility”, with the fields of ”memory and orientation” and ”inhibition” also showing lower performance. After surgery, patients presented an immediate worsening in their neuropsychological performance, particularly in the fields of “attention” and “flexibility”. Finally, we observed that some of the impaired neuropsychological capacities of the patients might improve some months after the surgery.

To summarize, our results suggest that: 1—pre-operative cognitive impairment is present in most patients; 2—immediate post-operative cognitive status shows an overall impairment, but only significant for attention and flexibility and almost significant for language; 3—at one-year follow-up, a secondary tendency towards improvement seems to be present in 5 out of 6 categories, but with a persistence of negative z-scores when compared to the standard population; 4—cognitive assessment is not standardized among patients; and 5—cognitive follow-up for meningioma patients remains short (approximately one year on average).

Several clinical neurosurgical series of olfactory groove meningiomas focus on surgical approaches (pterional, lateral supraorbital, fronto-lateral, subfrontal uni- or bilateral) and discuss their inherent advantages and disadvantages, frequently coming to the conclusion that experienced teams choose a standardized approach that becomes safer with practice [36,42]. Therefore, comparisons with regard to complication rates or postoperative deficits lack objective measurement and randomization. Among the reported outcomes, we commonly find KPS [7,8], recurrence rate [7,8], epilepsy [43], or olfaction [35], but implications in cognition remain underreported [5,7,8,36,42,44,45].

Because of the narrow space in the anterior fossa, and possibly the type of vascular supply to the tumor also coming from leptomeningeal branches of the internal carotid in this location, peritumoral brain edema was present more frequently in our series (82% with 35% of the brains presenting with an absence of a clear tumor–parenchyma delimitating interface on MRI, i.e., with the absence of a clear delimitation between tumor edges and healthy cerebral parenchyma) when compared to the existing literature [46]. The importance of pial blood supply in the development of peritumoral edema in meningiomas is well described. Of note, Delgado-Lopez et al. [47] recently reviewed causes for peritumoral edema, and according to their observations, the most important cause for edema seems to be the tumor size, which usually reaches a larger volume in this area compared to other locations, which is probably due to the late perception of cognitive symptoms. Furthermore, higher tumor grade and absence of a clear demarcation between the lesion and healthy parenchyma (which was particularly high in this cohort with 35%) also seemed to be associated with edema. All in all, this could contribute to a higher rate of cortical and subcortical damage and subsequent cognitive impairment. Nevertheless, olfactory groove meningiomas and their surgical treatment impacts 4 out of 6 domains of cognition, with a significant postoperative worsening of attention and flexibility (*p* < 0.05) (as well as a marked tendency towards significance for the category of language) and cognitive flexibility that persists even after a one-year follow-up.

Some previous studies provide insight into the cortical representation of these two domains of cognition that can help explain the bigger impact on cognitive flexibility [48,49,50]. Complex attention is a cognitive function that relies on the simultaneous, interconnected activity of several lobes (visual occipital cortex for awareness, afferent pathways, and regions of the parietal, and temporal and frontal lobes), thereby allowing for some plasticity that aids postoperative recovery [48]. In contrast, cognitive flexibility seems limited to medial areas of the pre-frontal cortex that perfectly match the affected regions in presence of olfactory groove meningiomas, which therefore explains the longer persistence of this deficit in task-switching or flexibility [22,27,28,49,50]. Cognitive flexibility is a major competence for coping with daily life requirements and, as such, a permanent deficit may fundamentally incapacitate one’s daily life in ways that are difficult to quantitatively assess and also to rehabilitate [51].

The present report has weaknesses inherent to every retrospective study. The sample size is small, and four patients did not have a complete neuropsychological assessment during follow-up and were excluded. Furthermore, the follow-up time was relatively short. As pointed out previously, the heterogeneity of assessment scales and measures used was significant, even within a single center and in a small neuropsychology team, and this weakness was only partially solved after categorization of each test in one of the six cognitive dimensions.

Our observations warrant the realization of further, prospective research to standardize neuropsychological assessment before and after surgical removal of an olfactory groove meningioma, especially by selecting adequate tests and time-points for neuropsychological examination and follow-up. The development of such a neuropsychological approach, specific (but not restricted) to the surgery of olfactory groove meningiomas, might lead to an improvement of the multi-modal management of such lesions. Indeed, early detection of neuropsychological deficits in patients might encourage quicker referral of the patients for specific rehabilitation therapy, allowing for further postoperative improvement in neuropsychological ability and resulting in a general improvement in quality of life. Finally, the development of such a neuropsychological assessment might also impact surgical or radiation therapy strategies by orienting the specialists towards a given surgical approach or radiation planning depending on the cognitive function of the cortical structures in direct contact with the lesion.

## 5. Conclusions

Olfactory groove meningiomas frequently present with edema and brain compression and therefore impact pre-frontal cortex cognitive functions. This impairment remains underreported and is more pronounced in the domain of cognitive flexibility, followed by attention. Updating seems the least affected by olfactory groove location. After an initial postoperative worsening, patients tend to improve in the majority of cognitive aspects, with attention and flexibility remaining impaired at one-year follow-up.

## Figures and Tables

**Table 1:** Patient and tumor characteristics. SD: Standard Deviation; WHO: World Health Organization.

Patients’ Age	Mean: 58.7 Years (Range 22–77)
Patients’ gender	Male: 9 (52.9%)
	Female: 8 (47.1%)
Tumor volume (mean +/− SD)	59 cm^3^ (+/−38.9)
Histological subtypes	Meningotheliomatous: 2 (12%)
	Transitional: 9 (52%) Meningothelial: 3 (18%)
	Psammomatous: 1 (6%) Atypical: 1 (6%)
	Not defined: 1 (6%)
WHO grade	I: 16 (94%)
	II: 1 (6%)
Preoperative perilesional edema on MRI	Yes: 14 (82%)
	No: 3 (18%)
Volume of preoperative perilesional edema	58.2 cm^3^ (+/−43.8)
(mean +/− SD)	
Absence of tumor–parenchyma delimitating interface on MRI	Yes: 6 (35%)
	No: 11 (65%)
Preoperative epilepsy	Yes: 4 (absence: 2, partial: 1, generalized: 1) (23%)
	No: 13 (77%)
Simpson resection grade	1: 4 (23%)
	2: 11 (65%)
	3: 1 (6%)
	4: 1 (6%)
Postoperative radiotherapy	Yes: 1 (6%)
	No: 16 (94%)
Neuropsychological follow-up	14 months (+/−5.7)
(mean +/− SD)	

## Data Availability

The datasets generated during and/or analyzed during the current study are available from the corresponding author on reasonable request.
